# Maternal autonomy and associated factors in making decision to utilize health service for themselves and neonates in south Ethiopia: A community based cross-sectional survey

**DOI:** 10.1371/journal.pone.0275303

**Published:** 2022-10-06

**Authors:** Degefa Gomora Tesfaye, Yohannes Tekalegn Efa, Fikreab Desta, Mulugeta Adugnew Gebeyehu, Sana’a Kedir Abdella

**Affiliations:** 1 Department of Midwifery, School of Health Sciences, Goba Referral Hospital, Madda Walabu University, Bale Goba, Ethiopia; 2 Department of Public Health, School of Health Sciences, Gobba Referral Hospital, Madda Walabu University, Bale Goba, Ethiopia; 3 Department of Nursing, School of Health Sciences, Gobba Referral Hospital, Madda Walabu University, Bale Goba, Ethiopia; Flinders University, AUSTRALIA

## Abstract

**Background:**

The definition of women’s autonomy used in the study is control over finances, decision–making power, and the extent of freedom of movement by women. Lower autonomy of women affects the socio-economic, emotional, fertility decision, contraceptive use, and sexual life of the women. Thus, this study aimed to assess maternal autonomy and associated factors in making a decision to utilize health services for themselves and neonates in south Ethiopia.

**Methods:**

Community-based cross-sectional study design was conducted from January 1 to March 2, 2021, in Shashamane town. Four hundred ten postpartum mothers were selected using a stratified random sampling technique and interviewed for the survey using questions composed of decision-making autonomy components (decision–making power, control over finances, and freedom of movement). The data were checked for consistency, coded, and entered using EpiData Manager (version 4.6.0.4) and analyzed using Statistical Package for Social Science (SPSS) version 26. Descriptive statistics, composite score analysis, and binary and multivariate logistic regression were done to capture the objectives.

**Result:**

410 postpartum mothers were interviewed while the mean and standard deviation of the participants’ age was 26.96 ± 5.38. About 48.5% of mothers had high decision-making autonomy for their own and their neonates’ health service utilization. Being in monogamous marriage (AOR = 1.82, 95% CI: 1.21, 2.74), and mode of delivery (Cesarean section) (AOR = 1.91, 95% CI: 1.18, 3.07) were significantly associated with having high maternal decision-making autonomy.

**Conclusions:**

More than half of the study participants had low maternal decision-making autonomy for their own and their neonates’ health service utilization. Being in monogamous marriage, and mode of delivery (Cesarean section) were factors significantly associated with high maternal decision-making autonomy. Encouraging mothers to use facility delivery was recommended.

## 1. Introduction

Different literatures used the definition of women’s autonomy as control over finances, decision–making power, and the extent of freedom of movement by women [13, 18–20, 23, 25]. Similarly, the definition of women’s autonomy used in this study was control over finances, decision–making power, and the extent of freedom of movement by women. Even though maternal autonomy is central to neonatal/child and maternal death prevention, it does not get more attention. Maternal mortality and child/neonatal mortality are major public health problems in low and middle-income countries. Evidence shows that adequate health service utilization and empowering women in decision-making about their health can improve maternal healthcare utilization [1–3].

The Lower autonomy of women affects the socio-economic, emotional, fertility decision, contraceptive use, and sexual life of the women. Notably, decisions made at the household level did not affect the welfare of the individual only. Further, it includes the surrounding community even at the country level. However, effective utilization of health services like early healthcare seeking can prevent the majority of neonatal death, that governed by decision-making power (autonomy) [4].

The effective utilization of health services like early care seeking can prevent the majority of neonatal death and be governed by decision-making autonomy. In many parts of the African country, there is poor early care seeking for neonatal care and child illnesses, as indicated by the death of home-delivered neonates. That is because the husband is the principal decision-maker than women and is poorly understood and considered women a neglected group in Ethiopia [5].

A report from the analysis of Indian demographic health survey conducted in 2015–16 years stated that utilization of maternal healthcare services was higher among women having a high level of decision-making autonomy in the household. However, no significant association was observed between women’s decision-making autonomy and institutional delivery in the adjusted analysis [6]. However, Afghanistan revealed that having high decision-making autonomy regarding control over finance (decision-making authority over how to spend their husband’s earnings) increased the likelihood of attending four or more ANC (antenatal care) visits, SBA (skilled birth attendance), and delivery by CS (Cesarean section) [7].

Analysis of the Nigerian Demographic and Health Survey (NDHS) reported that about 60% of women had low autonomy [8]. Moreover, study in Ghana showed that women have very little autonomy in deciding about their own and their neonates ‘and or child health service utilization. However, another study in Ghana revealed that 49.2% of the maternal and neonatal or child health service utilization was independently decided by husbands [9]. Decision-making autonomy of women is 46% and 52% in other parts of Ghana [10] and Uganda [11] respectively and it is low in Kenya (36%) [12].

Studies in different parts of the world showed that higher monthly income is associated with women’s autonomy for neonatal health care service utilization [13, 14]. Additionally, monogamous marriage [13], having an employed husband [13, 15] were positively associated with women’s autonomy while having a nuclear family was negatively associated [16]. Besides, studies from Iran and Nigeria found that older maternal age, exposure to mass media, higher socioeconomic status, higher educational status, higher family size, and knowledge of maternal and child health were positively associated with women’s decision-making autonomy [17, 18].

A similar finding was noted in parts of Ethiopia like in Ambo town, southern Ethiopia, and Bale zone Ethiopia found that older maternal age, exposure to mass media, higher socioeconomic status, higher educational status, higher family size, and knowledge of maternal and child health were positively associated with women’s decision-making autonomy [13, 19, 20].

In Ethiopia, only 11–18% of women were involved in making decisions alone, and 66–68% together with their husband or partner about their own and their neonates’ health service utilization [21]. In addition, about 41.4%) of women had higher autonomy regarding maternal and child or neonatal health care in other parts of Ethiopia [13]. Besides, about 75.1% of women in Debretabor had higher decision-making autonomy regarding their health, neonatal health, and other social and economic aspects [22].

Moreover, maternal decision-making autonomy for health care utilization was 55.6% in Ambo town and 58.4% in southern Ethiopia [19, 20]. Poverty, distance to health care services, and lack of education and awareness to use modern health care services, including reproductive health services, exacerbate the lowest level of autonomy [23].

Women in developing countries do not effectively use health care services for their neonates, which is indicated by low healthcare seeking for neonatal and child illnesses in resource-limited countries and the death of home-delivered neonates. Lack of decision-making autonomy was noted as a barrier not to utilizing health services effectively and timely. Because some women need to take permission from their husband/partners, or mother–in–laws before decisions on their health and that of their babies can be taken and could be late, or their spouses might not grant permission, especially if the spouse is not well educated [24].

In Ethiopia, specifically in the study area, there is a scarcity of study maternal autonomy and associated factors in decision making to utilize health services for themselves and neonates in south Ethiopia. In Ethiopia, a previous study did not emphasize neonatal danger signs, health care services, and decision-making autonomy among postpartum mother who was most commonly primary caregiver for their neonate. An early healthcare seeking is necessary to reduce neonatal mortality, while decision-making autonomy is essential, especially among caregivers or mothers. Thus, the objective of this study was to assess maternal autonomy and associated factors in making a decision to utilize health services for themselves and neonates in south Ethiopia.

## 2. Methods

### Study area, period and design

The community-based cross-sectional study design was conducted in Shashamane town, Oromia regional state, south Ethiopia from January 1 to March 2, 2021. The town is located 251 kilometers away from Addis Ababa, the capital city of Ethiopia. The town has eight kebeles (the smallest administrative unit in Ethiopia). The town has one general and one comprehensive specialized government hospital. Besides, one private hospital, four health centers, seventy-one medium private clinics, and seventy-two private pharmacies were health facilities in the town. The total town population was 279, 814 at the end of 2020 of which 141,150 were male and 138,665 were female [Shashamane town health office report, 2020].

### Population and eligibility criteria

All Postpartum mothers living in Shashamane town, Ethiopia during the study period were the source population while all randomly selected postpartum mothers who resided in the study area for at least six months and greater than or equal to 18 years old were included in the study and all mothers sampled happened to be married. All mothers who gave birth to stillbirth and are currently not with neonates were excluded.

Sample size determination and sampling procedure

The sample size was determined based on the single population proportion formula by considering the proportion of women who had higher autonomy scores in decision making regarding their health care, from the study conducted in Bale zone Oromia region 2014 [13] that was p = 41.4%, 95% confidence level, 5% marginal error(d), and 10% non-response rate. By considering these parameters, the final sample size became 410.

Postpartum mothers in the town were stratified by kebeles by using stratified random sampling techniques. The list of postpartum mothers with their addresses was prepared for each eight kebeles based on health extension workers’ registration data. The sample size was proportionally allocated to all kebeles based on respective number of postpartum women for all eight kebeles. Overall, using stratified random sampling by kebele and that sample size for each kebele was determined by the proportional representation mothers from that kebele in registration data (**Table 1**).

**Table 1:** Proportionally allocated sample of postpartum mothers in Shashamane town, Oomia, Ethiopia, 2021.

Kebeles	Average postpartum mothers in past two months	Proportionally allocated postpartum mothers
Kuyera	904	61
Abosto	900	60
Alelu	740	50
Bulchana	790	53
Arada	702	47
Dara Boqe	642	43
Awasho	778	52
Burqa Gudina	652	44

### Data collection tool, procedures, and data quality control

The questionnaire was prepared in the English language, contextualized to suit the research objective, local situations, and language, and translated to the local language (Afan Oromo) and back to the English language to check the consistency. Data was collected using a structured interviewer-administered questionnaire that was adapted after reviewing different pieces of pieces of literature [13, 14, 19, 20, 25–27].

The questionnaires were composed of sections such as socio-demographic characteristics, Health service uptake, Knowledge about Neonatal danger signs, Health care seeking practice, and Maternal decision-making Autonomy of maternal and neonatal health care services utilization. Four data collectors and 1 supervisor were involved in the data collection process. All of them have BSc degrees in midwifery.

The interview was conducted in the study participants’ homes. Pre-testing of the questionnaire was taken on 21(5%) of postpartum mothers in Goba town and Cronbach alpha > 0.7, training of the data collectors & supervisor, and close supervision of the data collection processes was considered to assure the quality of data.

#### Data processing and analysis

The data were checked for completeness and consistencies, coded, and entered using EpiData Manager (version 4.6.0.4). All supervisors were checked the completeness and correctness of the collected in daily bases. For data analysis and data cleaning, we used Statistical Package for Social Science (SPSS) version 26. The variables with p value less than 0.25 during binary logistic regression analysis were candidate for multivariable regression. The study utilized descriptive statistics, composite score analysis, and bivariable and multivariable binary logistic regression to capture the objectives. Hosmer-Lemeshow goodness of fit was done to test how well the data fits the model (p value = 0.60). Multicollinearity test was done to check whether there is inter-association or inter-relation between two or more independent variables is there or not. For this test VIF (Variance inflation factor was used and it is < 5) or an average VIF = 1.19).

Descriptive statistics were used to present socio-economic characteristics and other relevant characteristics of the women. A Composite score analysis was used to measure the level of maternal autonomy. Data was presented using texts, tables, graphs, and figures. Odds ratio at 95% confidence interval and p value less than or equal to 0.05 used for declaration of statistical significance.

#### Measurements

This study used the definition of women’s autonomy as the composite index of the three constructs of women’s autonomy: control over finance, decision-making power, and extent of freedom of movement [13, 18–20, 23, 25].

The index for maternal autonomy in this study was composed of fifteen total questions and categorized into three constructive components of women’s autonomy.

The first eight-question were used for addressing maternal decision-making power, four questions were used for addressing control over finance and the last three questions were used for addressing maternal freedom of movement [13, 28] (**S1 File**).

Responses to all these questions were measured using the following five responses that range from a maximum of four and a minimum of zero. Four are assigned if the decision is taken by the woman alone; three if the decision is taken by the woman and husband; two if the decision is taken by the woman and another person; one if the decision is taken by the husband alone; zero if the decision is taken by someone else. Then after recoded to one if women are involved either alone or jointly in decision-making and if not involved recoded to zero which means women are not involved either alone or jointly in the decision-making process. Regarding control over finance and freedom of movement assessing questions, there are seven Yes/ No questions. This is recorded in the way that 1 represents mothers had control over finance and had freedom of movement while 0 if the mothers’ responses to both questions of control over finance and freedom of movement of participants.

Adding those questions under decision-making power, control over finance, and freedom of movement components gives the maximum total score of 15 while the minimum score is 0 while the mean is 7.5. The overall maternal autonomy was classified as high decision-making autonomy if they score above the mean and Low decision-making autonomy when the score is below the mean using composite score analysis as used by other studies [13, 28].

### Operational definition

#### Fully immunized children

When they have received 1 dose of Bacillus Calmette Guerin (BCG), 3 doses of DPT (Diphtheria, Tetanus, Pertussis), 3 doses of polio vaccines and 1 dose of measles vaccination by the age of 9–12 months [29].

#### Not fully immunized

Children: when they missed at least one dose under the fully immunized group [29].

#### Post-Partum Mothers (PPM)

Mothers who gave births and within 6 months of period [30].

#### Postpartum period

A period with in six months after delivery and has three distinct phases (initial or acute period involves the first 6–12 hours postpartum, second phase is the sub-acute postpartum period, which lasts 2–6 weeks and third phase is the delayed postpartum period, which can last up to 6 months [30].

### Ethical considerations

Hawassa University College of medicine and health science institutional review board (IRB/094/12) granted ethical approval. All the study participants were assured about the anonymity of the data, informed about the purpose of the study, the variety of information needed from them, and informed that they were free to refuse or accept the interview. Informed verbal consent was taken from all study participants by reading the entire consent form for the study participants and the participants’ who were agreed to verbally read information sheet and sign the consent form to gave their permission were included in this study (S1 Appendix). Beside this, the data collectors continued with the interview process for study participants’ who were agreed to participate in the study.

## 3. Results

### Socio demographic characteristic of respondents’

Four hundred ten postpartum mothers were interviewed successfully and with a response rate of 100%. The mean and standard deviation of the participants’ age was 26.96 ± 5.38 while 182(44.4%) of the study participants were in the age group of 25–30. However, 210 (63.4%) participants had monogamy marriages, and 298 (72.7%) were unemployed (**Table 2**).

**Table 2:** Sociodemographic characteristics among postpartum mothers in Shashamane town, Oromia, Ethiopia, 2021. (N = 410).

Variables Category		Frequency	Percent
Mothers age	< = 24	138	33.7
	25–30	182	44.4
	31–35	57	13.9
	> = 36	33	8.0
Marriage type	Monogamy	210	51.2
	Polygamy	200	48.8
Ethnicity of mother	Oromo	279	68.0
	Sidama	47	11.5
	Guraghe	42	10.2
	Amhara	34	8.3
	Others^a^	8	2.0
Mother educational level	No formal education	122	29.8
	Primary	112	27.3
	Secondary school and above	176	42.9
Husband educational level	No formal education	96	23.4
	Primary	63	15.4
	Secondary school and above	251	61.2
Occupational status of mothers	Unemployed^b^	298	72.7
	Employed^c^	112	27.3
Husband occupational status	Unemployed	106	25.9
	Employed	304	74.2
Average monthly income	< = Median^d^	241	58.8
	> Median	169	41.2
Number of living children	< = 4	237	57.8
	> = 5	173	42.2

^a^ Wolaita, Silte, Kambata, Hadiya

^b^ Unemployed = daily labor, house wife, students

^c^ Employed = Merchant, government and Nongovernmental employed

^d^ Median average monthly income was 6000 ET Birr

### Health service utilization and decision making autonomy

Skilled birth attendants at health institutions (health centers, private and/or government hospitals) attended nearly eighty percent (326) of the respondents who resent baby delivery. However, only 147(35.9%) of the study participants had adequate ANC (antenatal care) follow-up (> = four visits). Apart from this, 298(72.7%) of the respondents had no postnatal follow-up, and 247(60.2%) of their current baby were received full immunization (fully immunized). The proportion (autonomous mothers) mothers who had high decision making autonomy among those who had adequate ANC(antenatal care) follow up (four and above ANC(antenatal care) visits), got ANC(antenatal care) counseling, got skilled birth attendance, had at least one postnatal follow-up, and fully immunized their current bay was 33.7%, 70.5%,29.1%,24.6% and 73.9% respectively (**Table 3**).

**Table 3:** Health service utilization and proportion of decision making autonomy among postpartum mothers in Shashamane town, Oromia, Ethiopia, 2021 (N = 410).

Variables	Category	Frequency	Percent	Decision making autonomy			
				Low		High	
				Frequency	Percent	Frequency	Percent
ANC(antenatal care) Follow up	No ANC follow up	109	26.6	42	19.9	67	33.7
	< 4 times visits	154	37.6	89	42.2	65	32.7
	> = 4 times visits	147	35.9	80	37.9	67	33.7
ANC(antenatal care)counseling service	No	92	30.6	53	31.4	39	29.5
	Yes	209	69.4	116	68.6	93	70.5
Skilled birth attendance	No	84	20.5	169	80.1	141	70.9
	Yes	326	79.5	42	19.9	58	29.1
Mode of delivery	SVD	310	75.6	82	38.9	81	40.7
	Cesarean section	100	24.4	129	61.1	118	59.3
Postnatal Follow up	No	298	72.7	148	70.1	150	75.4
	Yes	112	27.3	63	29.9	49	24.6
Attend immunization for their current baby	Not fully immunized	163	39.8	32	15.2	52	26.1
	Fully immunized	247	60.2	179	84.8	147	73.9

**Fig 1:**
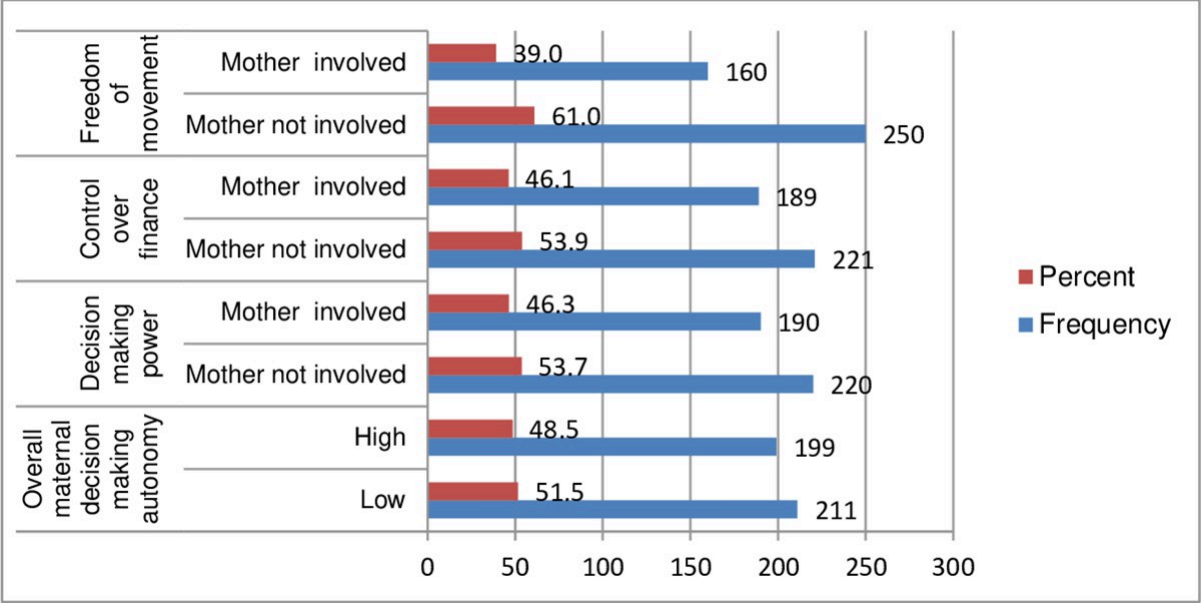
Maternal decision-making autonomy among postpartum mothers in Shashamate town, Oromia, Ethiopia, 2021 (N = 410).

The overall 48.5% (199) study participants had high decision-making autonomy for maternal and their neonates’ health care service utilization. However, About 190(46.3%), 189 (46.1%), and 160 (39%) of the study participants had decision-making power, control over finance, and Freedom of movement (**Fig 1**).

### Neonatal danger signs mentioned and practice by respondents

About 246 (60%) participants were able to mention at least one neonatal danger signs out of nine World health organization recognized neonatal danger signs. Meanwhile, 220(53.7%) of the respondents were initiated breastfeeding within one hour. of delivery, while 293 (71.5%) of the study participants fed colostrum to their current neonates’ (**Table 4**).

**Table 4:** Neonatal danger signs mentioned and practices done by postpartum mothers in Shashamane town, Oromia, Ethiopia, 2021.

Variables	Category	Frequency	Percent
Can you mention Neonatal Danger Signs and symptoms (N = 410)	No	164	40
	Yes	246	60
NDS mentioned by mothers (N = 246)	Unable to feed/poor feeding	182	74
	Fast breathing	146	59.3
	Hot to touch	135	54.9
	Convulsion	126	51.2
	Cold to touch	110	44.7
	Lethargy or weakness	104	42.3
	Umbilicus redness or draining pus, skin boils, or eyes draining pus	102	41.5
	Breathing difficulty	100	40.7
	Yellow skin or yellow soles	97	39.4
Practices of the respondents’ (N = 410)	Bathe their neonate after 24 hours. of birth	365	89.0
	Feeding colostrum for their neonate	293	71.5
	Initiate breast-feeding within 1 hour.	220	53.7
	Give pre lacteal fluid	86	20.9
	Applied substances on neonate cord	67	16.3

### Factors associated with maternal decision making autonomy

In bivariate analysis maternal education, maternal occupation, type of marriage, mode of delivery, initiated breastfeeding within 1 hour of delivery, and feeding colostrum for their current neonate were associated with having high maternal decision making autonomy. However, after adjusting for the above-listed variables (in multivariate analysis) monogamous mothers were 1.8 times more likely to have high decision-making autonomy when compared to polygamous mothers, AOR = 1.82 (1.21, 2.74). Having a low number of living children in the household is significantly associated with decision-making autonomy. Additionally, mothers who gave birth by cesarean section were 1.9 times more likely to have high decision-making autonomy when compared with mothers who gave births by spontaneous vaginal delivery AOR = 1.91(1.18,3.07) (**Table 5**).

**Table 5:** Factors associated with decision-making autonomy among postpartum mothers in Shashamane town, Oromia, Ethiopia, 2021.

Variables	Category	Maternal decision making Autonomy		COR,95%CI	AOR,95%CI
		Low	High		
		Count (%)	Count (%)		
Mother educational level	No formal education	55(26.1)	67(33.7)	1	1
	Primary	51(24.2)	61(30.7)	0.98(0.59,0.64)*	1.06(0.61,1.83)
	Secondary school and above	105(49.8)	71(35.7)	0.56(0.35,0.89)*	0.63(0.38,1.05)
Mothers occupation	Unemployed	143(67.8)	155(77.9)	1	1
	Employed	68(32.2)	44(22.1)	0.60(0.38,0.93)*	0.71(0.43,1.16)
Type of marriage	Monogamous	116(55)	84(42.2)	1.67(1.13,2.47)**	1.82(1.21,2.74)*
	Polygamous	95(45)	115(57.8)	1	1
Mode of delivery	SVD	169(80.1)	141(70.9)	1	1
	Cesarean section	42(19.9)	58(29.1)	1.66(1.05,2.61)*	1.91(1.18,3.07)*
Initiated breast feeding within 1 hr. of delivery	No	87(41.2)	103(51.8)	1	1
	Yes	124(58.8)	96(48.2)	0.65(0.44,0.97)*	1.16(0.66,2.02)
Fed colostrum for their baby	No	49(23.2)	68(34.2)	1	1
	Yes	162(76.8)	131(65.8)	0.58(0.38,0.90)**	1.34(0.72,2.48)

* = p value < = 0.05

** = p value < = 0.001, COR = crude odds ratio, AOR = Adjusted Odds Ratio

## 4. Discussion

This study assessed maternal decision-making autonomy and its determinants of health service utilization for their own and their neonates’ and found that 48.5% (95% CI: 43.7, 53.4) of the respondents had high maternal decision-making autonomy (mothers involved in the decision-making process of their own and their neonates’ health service utilization either alone or jointly with her husbands’). This finding is lower than the study done in Western Ethiopia (66.2%) [31], Northern Ethiopia (66.7%), and an Ethiopian demographic health survey 2016 (EDHS 2016) (74.4%) in which the study participants had autonomy in making health care decisions either alone or jointly with their husbands, respectively [19, 32].

Similarly, this study’s finding is lower than the report of the Ghana demographic health survey of 2014 that is about 75% of Ghanaian women had decision-making autonomy either alone or jointly with their husbands) [10]. However, this finding is slightly lower than the finding from the national family Health Survey (NFHS-4) India 2015–16 (53.6%) [6].

Besides this, this finding is lower than the study done in part of Ethiopia like Wollaita and Dawro zones (58.4%) [19],(Ambo town (55.6%) [20], Debretabor (75.1%) [22], analysis from the Ethiopian demographic health survey(EDHS) 2011 (54%) [33], Basoliben district (80%) [34] and further analysis of the EDHS 2016 reported that (81.6%) of women had higher decision-making autonomy [35].

The possible differences might be due to differences in the study participants’ socioeconomic characteristics, outcome variable measurement, study population, or sample size. On the other hand, our study uses different aspects to measure maternal decision-making autonomy like decision-making power, control over finance, and freedom of movement by using fifteen items unlike the study in Basoliban [34] and EDHS 2016 [35].

The other possible reason might be the difference in the educational status of mothers’ in which about 42.9% of study participants in this study had secondary and above educational levels. However, in the study done in Debretabor, 71.1% of women had secondary and above educational levels. On the other hand, the maternal occupational status might be the possible reason for the reason that this study’s finding was lower than that of the study’s conducted in Wolaita and Dawro. In this study, only about 27.3%, but in Wolaita and Dawro, 53% of the study participants were employed.

This study finding is higher than the Nigerian Demographic and Health Survey (NDHS) 2013 (40.5%) analysis [8], a study done in Bale zone Ethiopia (41.4%) [13], Nepal demographic health survey (37.9%) [36], Ghana (25.7%) [37], Nigeria (21.9%) [38]. The possible differences might be due to the difference in sociodemographic characteristics like our study include only urban participants while the study in Bale Nigeria, Ghana, and Nepal includes both rural in which utilization, accessing, and realizing of information is higher in the urban area. Another reason might be the difference in the definition of the outcome variable as noted in Nepal Demographic and Health Survey, 2011.

This study showed that monogamous mothers were 1.8 times more likely to have high decision-making autonomy when compared to polygamous mothers. This finding is consistent with the finding from Bale zone [13].

Moreover, mothers who gave birth by cesarean section were 1.9 times more likely to have high decision-making autonomy when compared with mothers who gave birth by spontaneous vaginal delivery. Similar findings were noted in elsewhere study [7]. This might be because autonomous mothers were more likely to give birth at health institutions than mothers who were not autonomous as stated in different studies were [39, 40] and given that cesarean delivery was done only in a health institution.

### Limitations of the study

Even though, this study utilized a composite score of three components called maternal decision-making power, control over finance, and freedom of movement to assess maternal decision-making autonomy. However, we have not assessed cultural barriers that might affect maternal decision-making autonomy. In addition, limitations due to being a cross-sectional study design (inability to identify causal association) were also considered a limitation of this study. In this study, we have not used a casually informed model.

## 5. Conclusion

More than half of the study participants” had low maternal decision-making autonomy deciding to utilize health services for themselves and neonates. Being in monogamous marriage and mode of delivery (giving birth by cesarean section) were factors that were significantly associated with having high maternal decision-making autonomy. Encouraging mothers to utilize facility delivery recommended. Meanwhile, health professionals and health extension workers should counsel each postpartum mother. Besides, strategies that can increase maternal or primary caregivers’ decision-making might be needed to reduce neonatal death.

## Supporting information

S1 File English version questionnaires for maternal autonomy. (DOCX)

S1 Appendix English version information sheet and consent form. (DOCX)

## Abbreviation and acronyms

ANC: Antenatal Care
AOR: Adjusted Odds Ratio
DPT: Diphtheria, Tetanus, Pertussis
NGO: Non-Governmental Organization
NMR: Neonatal Mortality Rate
PPM: Postpartum mother
