# Supplementary material for: Maternal autonomy and associated factors in making decision to utilize health service for themselves and neonates in south Ethiopia: A community based cross-sectional survey
Source: PLoS One. 2022 Oct 6;17(10):e0275303. doi: 10.1371/journal.pone.0275303 (PMC9536553; doi:10.1371/journal.pone.0275303)
Supplement: S1 File — (DOCX) [file pone.0275303.s001.docx]

**S1. Supplementary Information (English version questionnaires)**

**Part I: Socio-Demographic and Economic Data**

**1.1. Socio-Demographic Characterstics**

| **S. No** | **Variables** | **Alternatives** |
| --- | --- | --- |
| 101 | Age of mother | ______ years |
| 102 | Marriage Type | 1. Monogamy 2. Polygamy |
| 103 | Ethnicity mother | 1. Oromo 2. Sidama 3. Tigray 4. Amhara 5. Others (specifiy) ------------------------- |
| 104 | Religion of mother | 1. Muslim 2. Orthodox **Christian** 3. Protestant 4. Catholic 5. Others (specifiy)------------------------- |
| 105 | Educational status of mother | 1. No formal education 2. Elementary 3. Secondary &Preparatory 4. Certificate and above |
| 106 | Educational status of husband | 1. No formal education 2. Elementary 3. Secondary &Preparatory 4. Certificate and above |
| 107 | Occupational status of mothers | 1. Farmer 2. House wife’s 3. Merchant 4. Labor worker 5. Student 6. Employed (government/NGO) |
| 108 | Occupational status of husband | 1. Farmer 2. Merchant 3. Labor worker 4. Student 5. Driver 6. Other (specify….............) |
| 109 | Number of family members | _______ in numbers |
| 110 | What is your approximate household monthly income? | ________________ETB |
| 111 | Have you health insurance? | 1.Yes 2, No |
| 112 | Number of living children in house hold | 1. 1 2. 2. 3. 3 4. 4 5. More than 4 |

**II. Health Services Characteristics**

| **S.No** | **Variables** | **Alternatives** |  |
| --- | --- | --- | --- |
| 201 | ANC follow up | 1. Yes 2. No | If ‘NO ‘ skip to Q #204 |
| 202 | Number of ANC visits | ……………… times |  |
| 203 | ANC counseling services | 1. Yes 2. No |  |
| 204 | Have you received Skilled birth attendance for your recent child? | 1. No 2. Yes |  |
| 205 | Mode of delivery | 1. SVD 2. Cesarean section |  |
| 206 | PNC Attendance | 1. Yes 2. No |  |
| 207 | Is your recent neonate/child immunized | 1. No 2. Yes |  |
| 208 | If Yes for Q# 207, Is your neonate/child received: | 1. 1 dose of Bacillus Calmette Guerin (BCG), 2. 3 doses of DPT, 3. 3 doses of polio vaccines 4. 1 dose of measles vaccination by the age of 9–12 months 5. Other…………. | Ask by site of injection specifically for each vaccine and number of visit.  *Multiple answer possible* |

**Part III. Mothers’ recognition on neonatal danger signs and practices**

| S.No | **Variables** | **Alternatives** |
| --- | --- | --- |
| 301 | Can you able mention neonatal danger signs? | 1. Yes 2. No |
| 302 | If yes for Q # 301 Can you mention neonatal danger sign/s? | ……………….. …………………  ….……………….. ………………  ….……………….. ………………. |
| 303 | Where did you get information about neonatal danger signs? Multiple answers is possible | 1. Health care providers 2. Radio 3. Television 4. Family 5. Neighbor 6. Others |
| 304 | Have you ever practiced applying substance on neonate cord at your home? | 1. Yes 2. No |
| 305 | Have you ever given pre-lacteal fluid for your neonate at your home? | 1. Yes 2. No |
| 306 | Have you ever practiced feeding colostrum for your neonate? | 1. Yes 2. No |
| 307 | Have you ever bathed your new born after 24 hrs? | 1. Yes 2. No |

**Part IV: Maternal decision-making Autonomy.**

| **S. No** | **Variables** | | **Alternatives** |
| --- | --- | --- | --- |
| 401 | Who in your family usually has the final say on the: | Your own health care | 1. Someone else 2. Husband alone 3. Woman and another person 4. Woman and husband 5. Woman alone |
| 402 | Who in your family usually has the final say on the: | Your neonates’ health care | 1. Someone else 2. Husband alone 3. Woman and another person 4. Woman and husband 5. Woman alone |
| 403 | Who in your family usually has the final say on the: | Number of children to have | 1. Someone else 2. Husband alone 3. Woman and another person 4. Woman and husband 5. Woman alone |
| 404 | Who in your family usually has the final say on the: | Contraception use | 1. Someone else 2. Husband alone 3. Woman and another person 4. Woman and husband 5. Woman alone |
| 405 | Who in your family usually has the final say on the: | Use of  ANC follows up | 1. Someone else 2. Husband alone 3. Woman and another person 4. Woman and husband 5. Woman alone |
| 406 | Who in your family usually has the final say on the: | Preference of delivery site | 1. Someone else 2. Husband alone 3. Woman and another person 4. Woman and husband 5. Woman alone |
| 407 | Who in your family usually has the final say on the: | Use of PNC follows up | 1. Someone else 2. Husband alone 3. Woman and another person 4. Woman and husband 5. Woman alone |
| 408 | Who in your family usually has the final say on the: | Use of immunization services for your baby | 1. Someone else 2. Husband alone 3. Woman and another person 4. Woman and husband 5. Woman alone |
| **Question to address control over finance among participants** | | | |
| 409 | Had you regular access to a source of money including both wages earned and gifts or support from family | 1. No 2. Yes |  |
| 410 | If Yes for Q#409 Could spend that money without consulting anyone | 1. No 2. Yes |  |
| 411 | Could you able to give final decision on household purchases, how your partners’ earnings will be used either alone or with your husband | 1. No 2. Yes |  |
| 412 | Could you able to give final decision on how the money you earned either alone or with your husband | 1. No 2. Yes |  |
| **Question to address freedom of movement among participants** | | | |
| 413 | Could you able to leave the house without accompany of another adult. | 1. No 2. Yes |  |
| 414 | Could you able to go out to take a child to health facility | 1. No 2. Yes |  |
| 415 | Could you able to visit family or relative | 1. No 2. Yes |  |
