# Supplementary material for: Maternal autonomy and associated factors in making decision to utilize health service for themselves and neonates in south Ethiopia: A community based cross-sectional survey
Source: PLoS One. 2022 Oct 6;17(10):e0275303. doi: 10.1371/journal.pone.0275303 (PMC9536553; doi:10.1371/journal.pone.0275303)
Supplement: S1 Appendix — (DOCX) [file pone.0275303.s002.docx]

## S2. Appendix A: English version information Sheet and Consent form

**Information Sheet**

**Greeting**!

Good morning/afternoon, dear respondent! My name is________________ and I am data collector for the study being conducted in Shashamane town by instructor Degefa and others. You are randomly selected as a participant in this study and you kindly request to answer for questionnaires.

**Study Title:** – Maternal autonomy and associated factors in making decision to utilize health service for themselves and neonates in south Ethiopia: a community based cross-sectional survey.

**Risks**: the risks of being participating in this study are minimal, only taking few minutes from your time.

**Benefit-** at this moment you may not get any direct benefit by being involved in this study but the information you provide is very important to solve problem associated with maternal autonomy to seeking care for themselves and their neonate.

**Confidentiality**: I assure you that the information you are going to give me will be kept in secrete and your personal identifiers will not include.

**Rights**: Participation in this study is voluntary. You have the right to declare not to participate in this study and you have the right to withdraw from participating at any time.

**Contact address**: If there is any question or unclear idea any time about the study or the procedures, do not hesitate to contact and speak to principal investigators with cell phone number: **0909224919** or **Gmail** address **degefag21@gmail.com**

**Permission:** Your support and willingness in responding the questions will be very important for the success of this study, So that I need your cooperation to answer the questions that I am going to ask you. Are you willing to participate in this study?

1. No (thank you) 2. Yes (continue )

Thank you very much for your cooperation.

**Consent form**

I heard the information sheet concerning this study (or have understand verbal explanation) and I understand what will be required of me and what will happen to me if I take part in this study. I also understand that any time I may withdraw from this study without giving a reason and I am free to fill or not to fill the questions. Similarly, I understand that the data collected from me does not include personal identifiers and it was anonymous. Finally, I have signed bellow to show my free interest to participate in this study.

Participant’s signature ________________________ Date __________________

Participant’s signature certifying that the informed consent will be given written.

Data collector’s name ___________________

Data collector’s signature ____________________ Date ___________
